# Eip74EF is a dominant modifier for ALS-FTD-linked VCP^R152H^ phenotypes in the *Drosophila* eye model

**DOI:** 10.1186/s13104-023-06297-z

**Published:** 2023-03-06

**Authors:** Madeleine R. Chalmers, JiHye Kim, Nam Chul Kim

**Affiliations:** Department of Pharmacy Practice and Pharmaceutical Sciences, College of Pharmacy, University of Minnesota, Duluth, MN 55812 USA

**Keywords:** Neurodegeneration, Eip74EF, microRNA-34, VCP, ALS-FTD

## Abstract

**Objectives:**

In 2012, Liu et al. reported that miR-34 is an age-related miRNA regulating age-associated events and long-term brain integrity in *Drosophila*. They demonstrated that modulating miR-34 and its downstream target, Eip74EF, showed beneficial effects on an age-related disease using a *Drosophila* model of Spinocerebellar ataxia type 3 expressing SCA3trQ78. These results imply that miR-34 could be a general genetic modifier and therapeutic candidate for age-related diseases. Thus, the goal of this study was to examine the effect of miR-34 and Eip47EF on another age-related *Drosophila* disease model.

**Results:**

Using a *Drosophila* eye model expressing mutant *Drosophila* VCP (dVCP) that causes amyotrophic lateral sclerosis (ALS) and frontotemporal dementia (FTD), or multisystem proteinopathy (MSP), we demonstrated that abnormal eye phenotypes generated by dVCP^R152H^ were rescued by Eip74EF siRNA expression. Contrary to our expectations, miR-34 overexpression alone in the eyes with GMR-GAL4 resulted in complete lethality due to the leaky expression of GMR-GAL4 in other tissues. Interestingly, when miR-34 was co-expressed with dVCP^R152H^, a few survivors were produced; however, their eye degeneration was greatly exacerbated. Our data indicate that, while confirming that the downregulation of Eip74EF is beneficial to the dVCP^R152H^*Drosophila* eye model, the high expression level of miR-34 is actually toxic to the developing flies and the role of miR-34 in dVCP^R152H^-mediated pathogenesis is inconclusive in the GMR-GAL4 eye model. Identifying the transcriptional targets of Eip74EF might provide valuable insights into diseases caused by mutations in VCP such as ALS, FTD, and MSP.

## Introduction

Multisystem proteinopathy (MSP) is a dominantly inherited autosomal disease with differing degrees of severity, ranging in different combinations of inclusion body myopathy (IBM), Paget’s disease, frontotemporal dementia (FTD), and amyotrophic lateral sclerosis (ALS) in the same family members [1, 2]. Around 70% of MSP patients have a missense mutation in the gene encoding for valosin-containing protein (VCP) [3]. VCP is a molecular chaperone with a wide range of functions, some of which include ubiquitin-dependent protein degradation, nuclear envelope construction, and assembly of the Golgi apparatus and endoplasmic reticulum [4]. The age of onset for MSP patients ranges from late thirties to mid-fifties [2, 5].

Despite no readily available treatments, regulatory RNAs might be a potential therapeutic technique for degenerative diseases such as MSP [6, 7]. MicroRNAs (miRNA) are notable as they have a wide degree of functions and are essential for development, including cell differentiation and aging [8, 9]. Interestingly, Liu et al. [8] identified that microRNA-34 (miR-34) is involved in the aging process in *Drosophila melanogaster*. When miR-34 was knocked out, life spans were reduced, and aging deficits were present including an increase in misfolded proteins and accelerated neurodegeneration. When miR-34 was overexpressed, the lifespan of adult *Drosophila* increased. Therefore, miR-34 may potentially protect against neurodegeneration and extend the lifespan by reducing disease-associated protein misfolding. Indeed, they tested miR-34 against *Drosophila* expressing the polyQ domain of mutant Ataxin-3 (SCA3trQ78) causing spinocerebellar ataxia and found that inclusion formation was slowed, and neurodegeneration was suppressed [8]. Finally, they identified Ecdysone-induced protein 74EF (Eip74EF) as a downstream target of miR-34, mediating protective effects against aging and neurodegeneration. Eip74EF encodes E74A, a transcription factor implicated in ecdysone-mediated cell death, which is required for development but deleterious in adult *Drosophila* [8, 10]. Liu et al. demonstrated that miR-34 targets Eip74EF and decreases E74A levels, resulting in protective effects against aging and neurodegeneration [8]. Their findings provide an intriguing possibility that miR-34 or siRNAs against Eip74EF may have roles in other age-related degenerative diseases that involve protein misfolding and inclusion formation such as mutant VCP-induced MSP, ALS, and FTD. Importantly, the evolutionarily conserved miR-34 might also act as a genetic modifier in humans and may be a potential candidate for therapeutics. Therefore, we hypothesized that miR-34 and its downstream target, Eip74EF, are genetic modifiers for mutant VCP causing age-related degenerative diseases and tested this hypothesis briefly using a previously well characterized *Drosophila* eye model expressing dVCP^R152H^ by GMR-GAL4 [4].

## Methods

### Drosophila lines

All lines were obtained from the Bloomington Stock Center unless noted.

35785 y[1] sc[*] v[1] sev[21]; P{y[+ t7.7] v[+ t1.8] = VALIUM20-mCherry}attP2: siRNA for mCherry.

29353 y[1] v[1]; P{y[+ t7.7] v[+ t1.8] = TRiP.JF02515}attP2: siRNA for Eip74EF (FBgn0000567).

63790 w[*]; P{w[+ mC] = UAS-DsRed-mir-34.L}3/TM3, Sb[1] Ser[1]: mir-34.

41158 w[1118]; P{y[+ t7.7] w[+ mC] = UAS-LUC-mir-34.T}attP2: mir-34.

61386 w[*]; P{y[+ t7.7] w[+ mC] = UAS-mCherry.mir-34.sponge.V2}attP40; P{y[+ t7.7]w[+ mC]=.

UAS-mCherry.mir-34.sponge.V2}attP2/TM6B, Tb[1]: mir-34 sponge.

GMR-GAL4, UAS-dVCP^R152H^/CyO, and GMR-GAL4 were gifts from Dr. J. Paul Taylor.

### Fly maintenance

*Drosophila* were crossed and grown on Bloomington Stock Center standard cornmeal food at 25℃. Progeny were collected at two days old.

### Imaging technique

*Drosophila* eye pictures were taken using a Leica Z16 APO with the DMC2900 camera and a ring light.

### Statistical analysis

Eye phenotype quantification was performed with ilastik [11] and Flynotyper [12] as previously published [13]. Graphs visualizing the effect size (mean difference with 95% confidence interval) were generated with Estimation Stats, http://estimationstats.com [14]. Although we performed one-way ANOVA with post-hoc Dunnett’s tests, we omitted significance stars from our figure for more accurate evaluation of the data. However, p < 0.05 was still defined as significant to help facilitate the interpretation of the results.

## Results

To test our hypothesis, we examined genetic interactions between dVCP^R152H^ and miR-34 pathways. We used a previously published *Drosophila* model expressing mutant *Drosophila* VCP^R152H^ (dVCP^R152H^) in eyes with GMR-GAL4 [4]. This model has been successfully used to find genetic modifiers modulating mutant VCP pathogenesis [4, 15]. When dVCP^R152H^ was expressed in *Drosophila* eyes, severe eye degeneration was observed as published [4]. We examined the effect of miR-34 overexpression, miR-34 sponge, and RNAi-mediated knockdown for Eip74EF, using this rough eye phenotype. Based on the findings of Liu et al., we expected to see a rescue in the degenerative eye phenotype when Eip74EF had reduced expression, as well as a rescue in the eye phenotype when miR-34 was overexpressed. We also expected that miR-34 sponge might have no effect or exacerbate the rough eye phenotype slightly if miR-34 is expressed in the early developmental stage, as miR-34 sponge should reduce active miR-34.

**Fig. 1:**
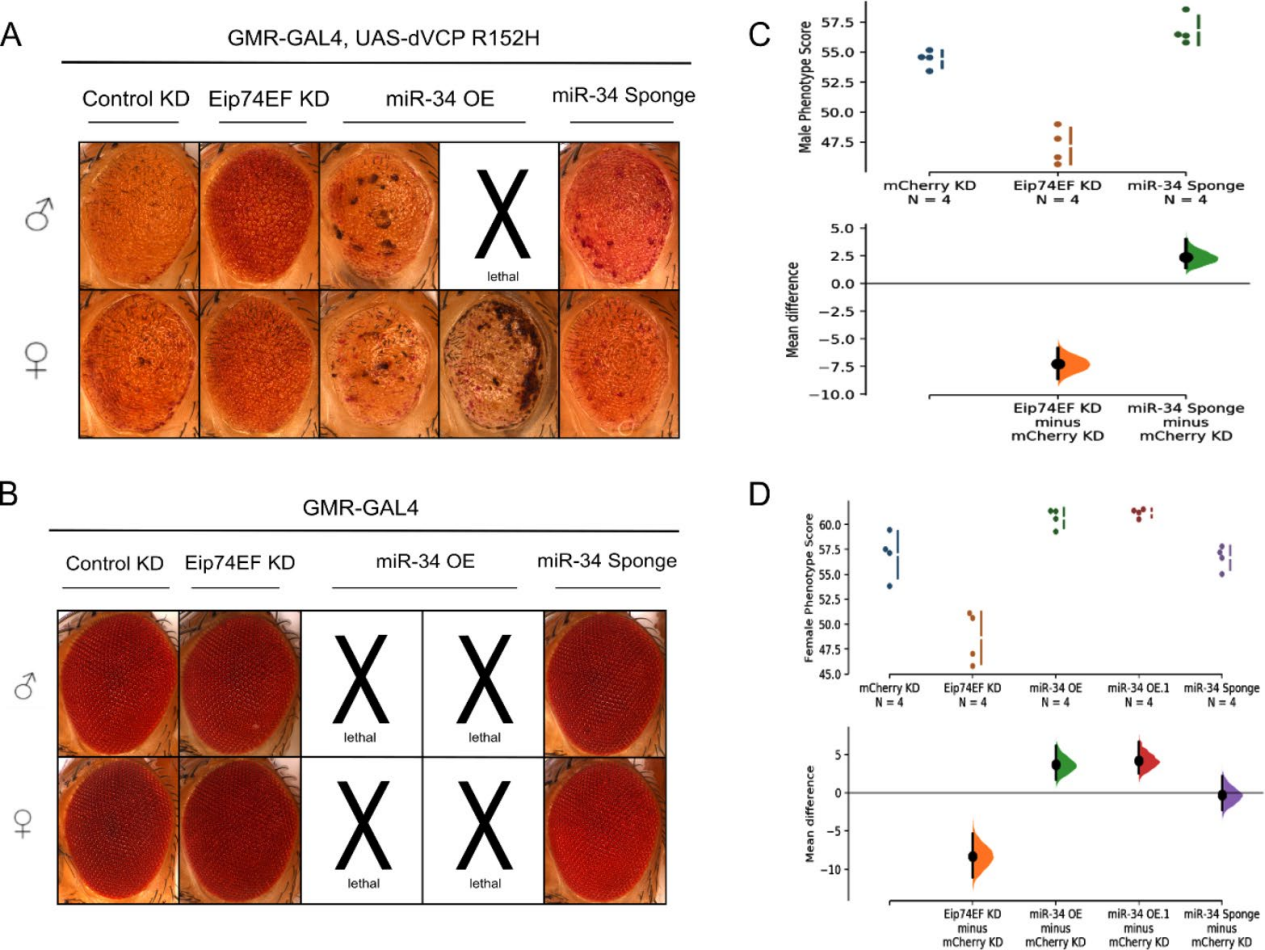
**Eip74EF siRNA significantly rescues degenerative phenotypes while miR-34 overexpression results in severe degeneration or lethality: (A)** Eip74EF siRNA expression (Eip74EF KD) significantly reduced dVCP^R152H^-induced abnormal eye phenotypes compared to mCherry siRNA expression (Control KD). miR-34 overexpression (OE) with dVCP^R152H^ resulted in severe eye degeneration or lethality. miR-34 sponge with dVCP^R152H^ resulted in slight enhancement in males (C) but did not show any effect in females (D). **(B)** Expression of transgenes with GMR-GAL4 to determine the lethality of the lines on their own. miR-34 OE lines were determined to be lethal, but mCherry siRNA, Eip74EF siRNA, and miR-34 sponge expression did not cause any effect on eyes. **(C)** Severity score quantification of male *Drosophila*. The raw data distribution is shown (upper panel) and the mean differences between control and experimental groups are plotted (lower panel) as dots with the vertical error bars indicating 95% confidence intervals. An independent statistical analysis was performed by One-way ANOVA with post-hoc Dunnett’s test (Control vs. Eip74EF siRNA: p < 0.0001, Control vs. miR-34 sponge: p = 0.037. Please note that there is no quantification for miR-34 OE males because there was N = 2, which was not enough for statistical analysis. **(D)** Severity score quantification of female *Drosophila*. One-way ANOVA with post-hoc Dunnett’s test (Control vs. Eip74EF siRNA: p < 0.0001, Control vs. miR-34 OE: p = 0.029 and 0.013, Control vs. miR-34 sponge: p = 1). Bloomington Stock Center lines from left to right: 35785, 29353, 63790 (miR-34 OE), 41158 (miR-34 OE.1), 61386

*Drosophila* expressing dVCP^R152H^ in their eyes (GMR-GAL4, UAS-dVCP^R152H^/CyO) were crossed with *Drosophila* lines carrying a construct for RNAi of Eip74EF (Bloomington 29353), miR-34 overexpression (Bloomington 63790, Bloomington 41158), and miR-34 sponge (Bloomington 61386). mCherry siRNA control line (Bloomington 35785) was used as a negative control. Eip74EF siRNA expression significantly rescued abnormal eye phenotypes generated by dVCP^R152H^ compared to the degenerated eyes of control siRNA (mCherry siRNA) expressing *Drosophila* in both males (Fig. 1A and C) and females (Fig. 1A and D). However, both lines of miR-34 overexpression induced either lethality or severe eye degeneration with dVCP^R152H^ (Fig. 1A). Due to the lethality caused by miR-34 with dVCP^R152H^, we only obtained 2 survivors for the male dVCP^R152H^ with miR-34 group in two independent experiments. Thus, it is not included in the graph and statistical analysis. miR-34 sponge expression slightly exacerbated the degenerative eye phenotype in males (Fig. 1A and C) but did not show any difference in females (Fig. 1A and D). This difference might be due to male-female difference or a technical limitation for rough eye phenotype quantification. As we did not expect severe degeneration or lethality among miR-34 overexpressing flies based on the previous publication [8], we crossed all tested *Drosophila* lines with GMR-GAL4 to determine if any of the lines used in this study were toxic on their own. Indeed, the two miR-34 overexpressing lines were completely lethal on their own with GMR-GAL4 due to their leaky expression in other tissues [16] (Fig. 1B), while a few survivors having severely degenerated eyes were produced with GMR-GAL4, UAS-dVCP^R152H^ (Fig. 1A).

Thus, contrary to our expectation, we did not observe rescuing effects of miR-34 in the dVCP^R152H^-induced eye degeneration model. However, Eip74EF siRNA significantly mitigated the toxicity of dVCP^R152H^ in *Drosophila* eyes.

## Discussion

While we can establish that downregulation of Eip74EF is protective in the dVCP^R152H^*Drosophila* eye model, we cannot definitively link miR-34 to dVCP^R152H^-mediated pathogenesis in the GMR-GAL4 eye model. These unexpected results have several possible explanations. We used UAS-miR-34 lines from the Bloomington Stock Center, not the same miR-34 lines created by Liu et al. [8]. These lines likely have different expression levels of miR-34 when it is expressed with GMR-GAL4. We suspect that the Bloomington miR-34 lines have a high expression level compared to Liu’s lines and resulted in pupal lethality. However, please note that RNAi-mediated knockdown of Eip74EF successfully rescued dVCP^R152H^-mediated toxicity. Thus, the higher expression of miR-34 with GMR-GAL4 may induce lethality via other genes, and probably in other tissues, regulated by miR-34, not Eip74EF.

As we described, miR-34 and dVCP^R152H^ co-expression did not cause complete lethality. This may indicate that there are more complex genetic interactions between miR-34 and dVCP^R152H^, or the survivors may have relative lower expression of dVCP^R152H^ and miR-34 compared to dVCP^R152H^ alone or miR-34 alone due to the phenomenon known as GAL4 dilution [17]. If this is due to the GAL4 dilution, the exacerbated eye phenotypes of survivors still suggests that miR-34 expression with dVCP^R152H^ enhances the degenerative phenotype because the low level of dVCP^R152H^ should result in less degenerated eyes if miR-34 is protective or does not interact with dVCP^R152H^. Further, the toxicity of dVCP^R152H^ expressed by GMR-GAL4 is high from the late pupal stage to the early adult stage which is regulated by the ecdysone-mediated transcriptional program. Therefore, it is not surprising that the reduction of Eip74EF, an ecdysone-activated transcription factor, involved in apoptosis, ameliorates dVCP^R152H^ toxicity. If we use a *Drosophila* model expressing dVCP^R152H^ in the late adult stage, we might observe the expected beneficial effects of miR-34 overexpression.

Together, our initial data suggests that Eip74EF is a strong genetic modifier for the dVCP^R152H^*Drosophila* eye model. Because of its lethality, we were not able to conclude that miR-34 is a genetic modifier. Although Eip74EF is not well conserved in humans, further studies identifying downstream transcriptional networks regulated by Eip74EF might provide valuable insights into what cellular pathways are involved in mutant VCP-caused diseases and how to treat these diseases.

### Limitations

While the data is meaningful in the *Drosophila* eye model and useful to other researchers in the Drosophila research community, our findings are primarily observational. To confirm these results, experiments using other *Drosophila* tissues such as muscle (MHC-GAL4) and neurons (ELAV-GAL4/Nsyb-GAL4) may be performed. In addition, we only observed siRNA-mediated phenotypic changes. Increased expression of Eip74EF or a validated human ortholog may provide further supporting evidence for our findings. Finally, we used different fly lines from previous literature claiming protective effects of miR-34 on neurodegeneration. Thus, further studies into the interactions between miR-34 and dVCP^R152H^ with different miR-34 lines may be necessary or a different eye-specific GAL4 line (Rh1-GAL4) may need to be used to avoid the lethality.

## Data Availability

The data are available from the corresponding author upon request.

## Abbreviations

ALS: Amyotrophic lateral sclerosis
FTD: Frontotemporal dementia
IBMPFD-ALS: Inclusion body myopathy with Paget’s disease, frontotemporal dementia, and amyotrophic lateral sclerosis
MSP: Multisystem proteinopathy
VCP: Valosin-containing protein
miR-34: microRNA-34
Eip74EF: Ecdysone-induced protein 74EF
